# In vitro model for investigating aerosol dispersion in a simulated COVID-19 patient during high-flow nasal cannula treatment

**DOI:** 10.3389/fmed.2022.1002659

**Published:** 2022-12-01

**Authors:** James A. McGrath, Andrew O’Sullivan, Mary Joyce, Miriam A. Byrne, Jie Li, James B. Fink, Ronan MacLoughlin

**Affiliations:** ^1^Department of Physics, School of Natural Science, Ryan Institute’s Centre for Climate & Air Pollution Studies, University of Galway, Galway, Ireland; ^2^Research & Development, Science & Emerging Technologies, Aerogen Limited, Galway, Ireland; ^3^Division of Respiratory Care, Department of Cardiopulmonary Sciences, Rush University Medical Center, Chicago, IL, United States; ^4^Aerogen Pharma Corporation, San Mateo, CA, United States; ^5^School of Pharmacy and Biomolecular Sciences, Royal College of Surgeons in Ireland, Dublin, Ireland; ^6^School of Pharmacy and Pharmaceutical Sciences, Trinity College Dublin, Dublin, Ireland

**Keywords:** COVID-19, bioaerosol, high-flow nasal cannula, dispersion, exposure, facemask, infectious disease, surgical mask

## Abstract

The use of high-flow nasal cannula in the treatment of COVID-19 infected patients has proven to be a valuable treatment option to improve oxygenation. Early in the pandemic, there were concerns for the degree of risk of disease transmission to health care workers utilizing these treatments that are considered aerosol generating procedures. This study developed an *in vitro* model to examine the release of simulated patient-derived bioaerosol with and without high-flow nasal cannula at gas flow rates of 30 and 50 L/min. Aerosol dispersion was evaluated at 30 and 90 cm distances. Reduction of transmission risk was assessed using a surgical facemask on the manikin. Results indicated that the use of a facemask facilitated a 94–95% reduction in exhaled aerosol concentration at 30 cm and 22–60% reduction for 90 cm distance across both gas flow rates. This bench study confirms that this *in vitro* model can be used as a tool to assess the risk of disease transmission during aerosol generating procedures in a simulated patient and to test factors to mitigate the risk.

## Introduction

With the onset of the COVID-19 pandemic, the options for inhalation drug therapies and vaccines (1) were unknown, leading to the sole reliance on respiratory support interventions for treatment. One such intervention was High-flow nasal cannula (HFNC), which has shown to improve oxygenation and reduce the need for intubation for hypoxemic patients (2). Early in the pandemic oxygen administration via HFNC was identified as one of many aerosol generating procedures (AGPs) suspect in the transmission of SARS-CoV-2 virus to health care workers (HCWs), resulting in institutions abandoning HFNC as a tool for treatment of hypoxia and respiratory distress for suspected COVID-19 patients, in favor of early intubation and mechanical ventilation for many patients who would otherwise have benefited from HFNC (3). This created a high demand for critical care ventilators producing an international shortage (4, 5). Evidence suggests that HFNC can reduce the need for mechanical ventilation by as much as 71% in COVID-19 patients (6). So further evaluation was warranted to identify options to reduce risk associated with this important form of therapy.

Multiple efforts to quantify increased virus transmission of patient generated bioaerosols during HFNC have failed to establish a procedure-associated increase in the level of transmission (7). However, the application of oxygen is associated with increased dispersion of aerosols generated by the patient rather than increased generation (8).

Compared to breathing activities with room air or conventional nasal cannula, HFNC did not generate higher particle concentrations than normal breathing, while cough or deep breathing generated more aerosol particles (9, 10). Placing a surgical mask over a nasal cannula has been advocated, regardless of the gas flow settings, to reduce dispersion of patient generated bioaerosols (11, 12). Having patients wear surgical masks can reduce the risk of transmission to HCWs (13). This has been reported in healthy subjects (14) as well as COVID-19 patients (15). Reduced dispersion has been confirmed in silica, with computational fluid dynamic simulations reporting that wearing a surgical/procedure mask over HFNC may reduce aerosol droplet dispersion (16). Many of the currently published studies utilized human beings (healthy volunteers or patients) to investigate the aerosol particle dispersion and the effects of mitigation strategies, however, implementation of those studies is time consuming and difficult to control breathing patterns and ambient variables, more importantly, it may incur unnecessary infection risk for study investigators. Therefore, building a more representative *in vitro* model may help resolve this dilemma. Previous studies placed a continuous jet nebulizer at the manikin’s trachea to simulate aerosol particle dispersion or assess transmission risk, however, the continuous production of aerosol is not representative or realistic, as human subjects would not disperse aerosols during inhalation phase. Thus, we developed a model with an aerosol generator that emits aerosol synchronized with expiration placed in the manikin’s trachea, to simulate the production of bioaerosol during natural exhalation. This study aimed to characterize the aerosol dispersion with and without HFNC treatment using this *in vitro* model, as well as the effects of wearing a surgical mask over HFNC.

## Materials and methods

### Anatomical model

A previously described airway model of the adult nose–throat region was used as the adult model (17).

### High flow nasal therapy circuit and interface

A humidified high flow system (Airvo 2, Fisher & Paykel Healthcare, Auckland, New Zealand) was used with an adult breathing circuit (P/N: 900PT552) and nasal cannula (P/N: OPT 944). The Airvo 2 HFNC system features a humidifier with an integrated flow source. All testing was completed at clinically relevant gas-flow rates (30 and 50 L/min HFNC for simulated adult) (2, 15, 18, 19).

A medium adult nasal cannula was positioned in the nose of an anatomically correct nose–throat adult model, in accordance with manufacturers’ instructions. The nose–throat model was connected to a breathing simulator (ASL 5000, Ingmar Medical, Pittsburgh, PA, USA) via a hydrophobic filter (HF) (Pall Breathing Circuit Filter BB-50T; Pall Biomedical Products, Port Washington, NY). The breathing simulator generated a hypoxemic adult breath pattern (tidal volume 700 mL, breath rate 25 BPM and inspiratory: expiratory ratio 1:1.5) for each test condition in this study. A novel breath actuated aerosol generator (20, 21) was placed between the filter and the trachea of the head model and set to generate simulated bioaerosol on the peak expiratory flow during the exhalation phase of each breath to simulate a patient exhaling. Normal saline (0.9%, BRAUN, Dublin, Ireland) was nebulized as a simulated bioaerosol.

### Experimental test setups

**FIGURE 1:**
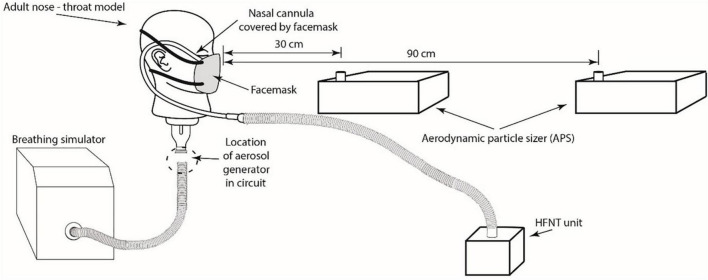
Illustration of an *in vitro* model with HFNC interface and surgical facemask with the APS positioned at 30 and 90 cm distance relative to the head model. Note, test combinations were examined with and without a standard surgical facemask on the adult nose-throat model.

In total, six different test combinations were examined; the aforementioned gas flow rates of 30 and 50 L/min for high flow treatment, and a third set up, as a control, with the simulated patient breathing independently of the high flow system. These three test combinations were then examined with and without a standard surgical facemask worn by the head model (Figure 1).

### Bioaerosol characterization

The Aerodynamic Particle Sizer (APS) (APS, model 3321 TSI Inc., St. Paul, MN, USA) was used to characterize aerosol concentrations. Throughout the experiments, the APSs continuously measured particle number concentrations (PNC) and size distributions (0.5–20 μm) of the airborne concentration in the room. Two APSs, located at a distance of 30 and 90 cm, were positioned directly in front of the head model (Figure 1). The 30 cm distance was chosen as it simulated a caregiver fitting nasal cannula onto a patient’s face receiving the HFNC. The distance of 90 cm was selected as the vicinity of care givers performing less direct care procedure to the patient (15).

The APSs were set to record data at five second intervals for a total of 10 min. An initial two-minute period established a baseline measurement of ambient aerosol in the room prior to activating the aerosol generator. The remaining eight minutes monitored simulated bioaerosol being released on exhalation from the simulated breathing patient. After each test, the room was ventilated, and the aerosol concentration was monitored until it returned to ambient levels (16 ± 4 cm^3^). In order to focus on the exhaled aerosol, the PNC from two-minute baseline measurements was subtracted from all the time-series data, therefore, all the data present here reflects the exhaled simulated bioaerosol from the simulated patient.

### Temperature and airflow characteristics

The room in which the study was conducted had dimensions L = 6.85 m, W = 3.42 m and H = 2.50 m. The air change rate was measured using the tracer gas decay method with CO_2_ as the tracer, and a GrayWolf probe IQ-610 (GrayWolf Sensing Solutions; Shelton, CT, USA) was used for gas detection. The air change rate was calculated to be approximately 1.15 h^–1^. Room temperature in the laboratory room were measured using a digital hygro-thermometer-datalogger (Fisherbrand, Thermo Fisher Scientific, Massachusetts, USA) and values recorded were in the ranges 19.4 to 20.9°C. The experiments took place over a two-day period.

### Data analysis and statistics

The data analysis for this study was performed using the statistical package IBM SPSS Statistics25 (IBM Corp., Armonk, NY, USA, 2013). Summary and descriptive statistics were performed on the exhaled bioaerosol PNC. All distribution characteristics are summarized by arithmetic mean and standard deviations. Due to the non-parametric nature of the data, the Mann-Whitney test was performed to test for significance between mask and unmasked test scenarios. Differences between runs were considered statistically significant when *p* < 0.05.

## Results

**FIGURE 2:**
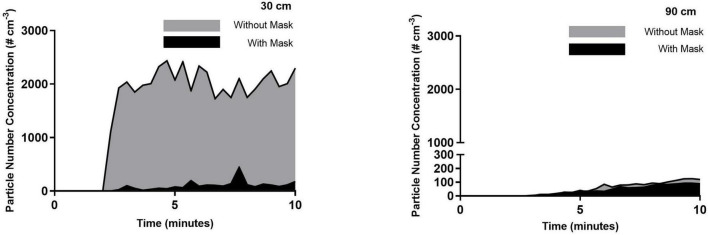
Simulated exhaled aerosol with no HFNC, with and without a mask (control). Averaged time-series PNC for the three runs recorded at a distance of 30 and 90 cm from the simulated patient. Each trendline represents the data averaged across 20 s intervals.

**FIGURE 3:**
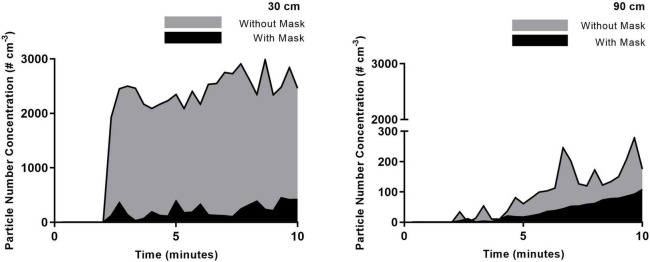
30 L/min HFNC with and without mask. Averaged time-series PNC for the three runs recorded at a distance of 30 and 90 cm from the simulated patient. Each trendline represents the data averaged across 20 s intervals.

**FIGURE 4:**
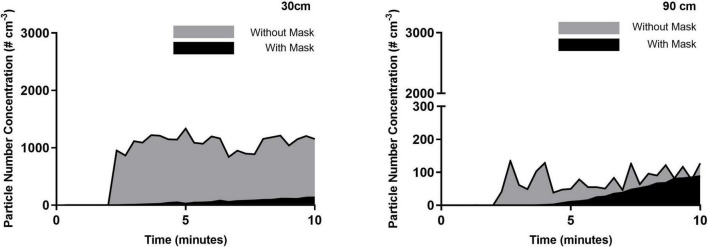
50 L/min HFNC with and without mask. Averaged time-series PNC for the three runs recorded at a distance of 30 and 90 cm from the simulated patient. Each trendline represents the data averaged across 20 s intervals.

Figures 2–4 highlight the initial two minutes of ambient aerosol measurements followed by the increases in aerosol concentration released by a simulated patient under all six scenarios. Table 1 shows a clear distinction between the scenarios where the patient is wearing/not wearing a facemask. When the patient was wearing the facemask, there was a 94–95% reduction in exhaled aerosol concentration at a distance of 30 cm from the simulated patient, compared to the no-facemask scenario for all three high flow rates (*p* < 0.0001). At a distance of 90 cm from the simulated patient, this reduction decreased to 22, 60, and 58% for the no nasal cannula (no high flow), 30 and 50 L/min scenarios respectively (*p* = 0.4084, 0.0201, 0.0053).

**TABLE 1:** Ten-minute averaged PNC during each high flow rate; mean and standard deviations over the three events.

Test	Distance	No high flow (# cm^–3^)	30 LPM (# cm^–3^)	50 LPM (# cm^–3^)
With a mask Without mask	30 cm 30 cm	83 ± 24 1612 ± 40	96 ± 12 1953 ± 338	51 ± 14 877 ± 88
With a mask Without a mask	90 cm 90 cm	38 ± 11 49 ± 11	35 ± 6 88 ± 25	27 ± 4 64 ± 19

There is an observed increase in aerosol concentrations at the 30 cm distance within the first 20 s of the experimental run. Within 40 s, the peak concentrations are observed, and while there are fluctuations over the following seven minutes, there are no substantial changes; this highlights the ongoing exhalation of aerosol from the patient. Within this distance, the plume released from the patient maintains a stable aerosol concentration over the sampling duration, indicating that accumulation is not occurring within the 30 cm radius but instead the aerosol is dispersing throughout the room. Measured aerosol concentrations at a distance of 90 cm confirm this finding. At this distance, the aerosol concentrations are typically reduced by one order of magnitude, which can be explained by considering the dilution due to increased volume ratio. However, in contrast with the measurements at the 30 cm, the aerosol concentrations at a distance of 90 cm were seen to increase over the 2–10 min period of the experimental run, demonstrating an accumulation in this zone over the sampling duration. The influence of the high flow from the nasal cannula interface is evident at the greater distances from the patient, as seen in 90 cm data, where the highest initial aerosol concentration corresponds to the 50 L/min flow rate, followed by 30 L/min, for the unmasked scenario. There is a slower accumulation of aerosol when there is no nasal cannula interface present, due to the slower velocity as it is not increased by the high flow setup. Comparing the data shown in Table 2 and the 90 cm data, it is worth noting that a greater reduction in the time-weighted average concentration is seen in the masked scenarios; by contrast, however, the 90 cm data highlights comparable aerosol concentrations for five scenarios at the 10-min mark suggesting a possible time-lag in the masked scenarios. It is suggested that the mask may reduce the velocity of the exhaled aerosol, creating a time-lag effect.

**TABLE 2:** The max PNC of each run; mean and standard deviations over the three events.

Test	Distance	No high flow (# cm^–3^)	30 LPM (# cm^–3^)	50 LPM (# cm^–3^)
With a mask Without mask	30 cm 30 cm	449 ± 221 2857 ± 165	295 ± 16 3466 ± 524	151 ± 47 1440 ± 49
With a mask Without a mask	90 cm 90 cm	105 ± 21 133 ± 14	115 ± 13 464 ± 126	90 ± 11 232 ± 109

## Discussion

The study design allows the opportunity to disentangle the influence of multiple external factors (e.g., varying occupant generation/breathing rates, room conditions, background sources) in real-world situations, which allows this approach to be used as an experimental model to investigate aerosol transmission risk. In this study, the application is demonstrated by using a breath-synchronized aerosol generator to provide a reproducible exhaled generation rate, which offers the ability to solely quantify the reduction provided by a surgical mask under different gas flow rates.

While the absolute PNCs reported in the current work were not generated by a real patient, they are reflective of data reported by Li et al. (15) from a clinical setting with confirmed COVID-19 patients: the unmasked patient data in the 0.5–10 μm size range at 1 foot (∼30 cm) recorded an average of 4,232 #/cm^3^ compared with averaged concentrations of 2,031–2,456 #/cm^3^ in the current work. Therefore, the data can be extrapolated to provide valuable insight into the relationship between the different high flow rates and distances traveled by emitted aerosol.

When the simulated patient was wearing a surgical mask during HFNC treatment, a reduction in aerosol concentrations was evident and reaffirms the use of face masks as a mitigation tool. Similar findings were reported from a study using computational fluid dynamics (CFD) (Leonard et al. (16)), which reported an 83.2% reduction in particles at 40 L/min under high-velocity nasal insufflation comparable with the 94–95% reduction observed in the present work. Similarly, the work showed that the majority of the particles that escaped traveled greater than 1 m, which is reflective in the results of the current work.

The experimental model allows a direct comparison between mask and no mask scenarios, under different high flow rates. The model shows that 30 L/min results in higher concentrations at a distance of 30 cm from a patient. In the masked scenario, the measured PNCs at the 30 cm mark are doubled for the 30 L/min compared with no HFNC (0 L/min). Although when the HFNC operates at 50 L/min, the concentrations are lower than no HFNC. This trend is consistent with the work of Gaeckle et al. (22), where measured PNCs were lower at 50 L/min compared with 30 and 10 L/min. It is possible that the lower gas flow rates provide enough of a driving force to push the aerosol through, or around, the mask, but as the air flow increases, it results in greater impaction on the mask and face. The model demonstrates that there are factors influencing aerosol dispersion which are independent of the patient.

Table 3 outlines the average % reduction in aerosol dispersion using a mitigating measure during HFNC reported in previous *in vivo* studies (14–16, 23, 24) in comparison to this current study using an experimental *in vitro* model. In contrast to this current study, there were no significant decreases in PNCs with the use of a facemask to mitigate against aerosol dispersion during HFNC at 50 L/min in an *in vivo* study by Li et al. (25). The participants of this study were healthy adults in comparison to this current study which utilized a simulated breathing pattern with a higher tidal volume and breath rate to mimic respiratory distress. Similar to a visualization study by Takazono et al. (14), which reported that the use of a surgical mask led to a reduction in particle dispersion by 80–95% during 30 and 60 L/min HFNC when speaking or coughing. However, they did not find significant reduction by wearing a surgical mask during HFNC while rest breathing. This was also in agreement with Hamilton et al. (23), who outlined that during HFNC, the use of a facemask reduced the reported aerosol concentrations during exertional respiratory efforts, i.e., coughing. Wilson et al. (24) findings also demonstrated that the use of a facemask led to a larger reduction in aerosol emissions in activities that required greater exertion. These *in vivo* studies have shown similar reductions in aerosol dispersion using a mask during distressed breathing/exertional respiratory activities, validating the potential use of this *in vitro* model to assess aerosol transmission risk during other respiratory support treatments in patients with varying clinical conditions.

**TABLE 3:** Comparison of average % reduction in aerosol dispersion using a mitigation measure during HFNC.

Publication	Detection method	Population	Mitigation measure	HFNC (LPM)	% Reduction
Current study	Particle sizer	Simulated distressed adult model ×1	Surgical mask	30 50	94–95
Takazono et al. (14)	Particle sizer	COVID-19 adult patient ×9	Surgical mask	50	60
Milton et al. (13)	Particle visualization system	Healthy adult volunteer ×5	Surgical mask	30 60	80–95
Gaeckle et al. (22)	Particle sizer	Healthy adult volunteer ×25	Surgical mask	30 60	80–86
Hamilton et al. (23)	Particle sizer	Healthy adult volunteer ×10	Surgical mask	60	70–80
Li et al. (15)	CFD	Simulated adult model ×2	Surgical mask	40	83

The dispersion of aerosol beyond 90 cm has been measured in other studies (26) and reported to be affected by various environmental and localized conditions; relative humidity, ventilation, room dimensions etc. The current model allows the ability to measure the relative contribution of each of these parameters independently and assess their overall influence. Lower concentration measurements at 90 cm are consistent with prior reports and is likely due in part to the dispersion of generated aerosol into the greater volume of air between the 30 and 90 cm boundaries, compared to the ambient background measurements at both distances. At these lower concentrations, at 90 cm the ambient aerosol have a greater influence over the reduction of percentages between the tested conditions.

The results highlight the need for multiple approaches to reduce the potential transmission of SARS-Cov-2. A surgical mask reduces the concentration in the near field, but appropriate ventilation appears to be needed to mitigate longer-range transmission (27).

## Conclusion

In conclusion, this experimental model highlights the ability to investigate aerosol transmission and dispersion, during HFNC and characterizing mitigation measures such as the application of a surgical facemask. The addition of a surgical facemask on a COVID-19 patient undergoing HFNC can reduce the quantity of patient-derived bioaerosol released into the surrounding environment. This model would be beneficial for further aerosol dispersion studies utilizing different respiratory support therapies and varying breath patterns which would include incremental validation against real-world data to aid in establishing clinical guidance. An incremental validation approach would ensure that the ability to disentangle the influence of multiple external factors remains a core strength of the current work. This model can be used to establish best practice for the use of this vital first line treatment for COVID-19 patients to prevent an escalation of care to more invasive ventilatory supports whilst mitigating the risk of disease transmission.

## Study limitations

Our testing was conducted using only one breathing pattern which was selected to characterize an adult with moderate respiratory insufficiency. This pattern of distressed breathing may not be representative of the possible range of different breathing patterns.

## Data availability statement

The raw data supporting the conclusions of this article will be made available by the authors, without undue reservation.

## Author contributions

JM, AO’S, and MJ conducted testing, data analysis, and manuscript draft. MB conducted data analysis. JL and JF provided guidance on experimental design and manuscript final review. RM designed experiments, conducted testing, manuscript draft, and final review.
